# A304 EXPLORING HEPATITIS C TREATMENT COST TRENDS OVER A DECADE: A CROSS-SECTIONAL ANALYSIS WITHIN MEDICARE PART D (2012-2021)

**DOI:** 10.1093/jcag/gwad061.304

**Published:** 2024-02-14

**Authors:** C H Tsai, G Malik, S E Congly

**Affiliations:** University of Calgary, Calgary, AB, Canada; University of Calgary, Calgary, AB, Canada; University of Calgary, Calgary, AB, Canada

## Abstract

**Background:**

Chronic hepatitis C virus (HCV) infection negatively impacts quality and quantity of life. With the onset of direct-acting antivirals (DAAs), cure rates of HCV have increased to over 95%. Despite these advancements, chronic HCV infection continues to pose a substantial health and financial burden and without drug coverage, treatment of HCV with these DAAs is unaffordable. In the United States, Medicare Part D is a voluntary drug coverage plan predominantly for individuals covered by Medicare (typically over 65 years old) which can facilitate access to these DAAs.

**Aims:**

This study aims to analyze the shifts in HCV treatment and spending in the USA from 2012 to 2021 for Medicare Part D and assess the impact of generic drugs on overall expenditure.

**Methods:**

A retrospective cross-sectional study was conducted using publicly available Centers for Medicare and Medicaid Services (CMS) Medicare D drug spending data from 2012 to 2021. The total expenditure and utilization of HCV drugs was calculated, and average spending per beneficiary was utilized to estimate potential cost savings if generic DAAs were employed.

**Results:**

The study revealed a decline in HCV claims and beneficiaries over 65 since 2015 with a peak of 134,752 beneficiaries decreasing to 35,735 in 2021. Accordingly, spending was the highest in 2015 (8.8 billion USD) and subsequently trended downwards to 1.5 billion in 2021. Over the ten-year period, the USA spent a total of 33.1 billion USD on HCV treatment. Harvoni accounted for 53% of the spending, followed by Sovaldi (17%) and Epclusa (14%). Generic drug utilization slowly increased with the introduction of generics in 2019 with 6.4% of beneficiaries treated with generic DAAs in 2019, rising to 15.3% in 2020 and 14.8% in 2021. If all prescriptions of Harvoni and Epclusa were substituted by ledipasvir/sofosbuvir and sofosbuvir/velpatasvir, respectively, there is a potential $2.7 billion cost reduction. This translates to a potential 47.3% reduction in HCV treatment expenditures from 2019 to 2021.

**Conclusions:**

The decreasing number of beneficiaries for HCV treatment among people over the age of 65 suggests a decreasing HCV prevalence in this age group. This reflects the success of DAA treatment and low reinfection rates. However, the underutilization of generic DAAs despite their lower cost highlights a missed opportunity for substantial savings. This study emphasizes the importance of policy negotiations to ensure optimal resource management, advocating for a shift towards generic drug usage to alleviate the financial burden associated with HCV treatment.

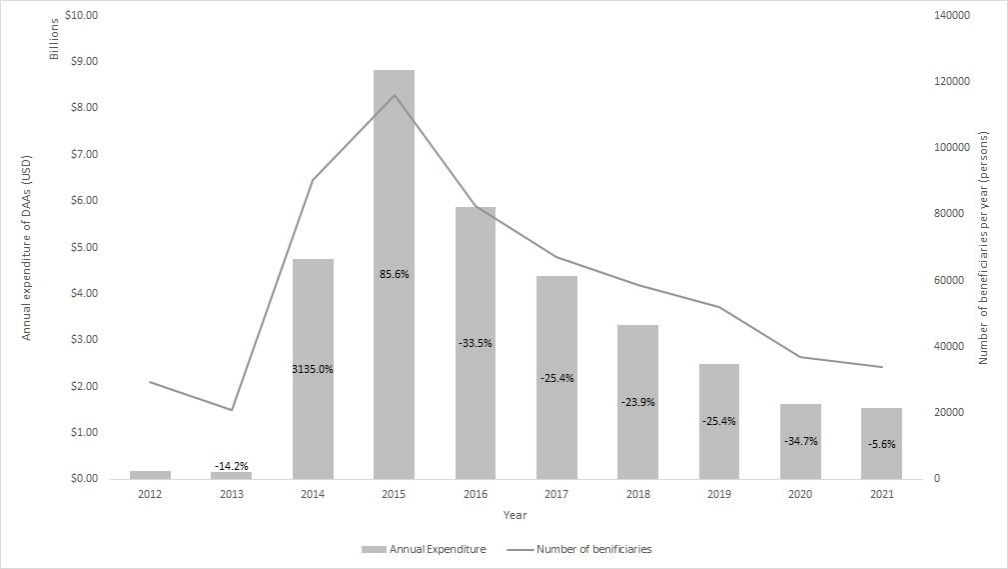

Trends in total spending on DAAs for HCV and number of beneficiaries within Medicare Part D from 2012 to 2021. Annual percent changes in expenditure are shown within the data bars.

**Funding Agencies:**

None

